# Editorial: Pediatric myeloid neoplasms: new insights into diagnosis, prognosis, and treatment

**DOI:** 10.3389/fped.2025.1604478

**Published:** 2025-04-29

**Authors:** Viviane Lamim Lovatel, Jeffrey J. Pu, Teresa de Souza Fernandez

**Affiliations:** ^1^ Cytogenetic Laboratory, Cell and Gene Therapy Program, Instituto Nacional de Câncer, Rio de Janeiro, Brasil; ^2^ Department of Medicine, VA Boston Medical Center and Brigham & Women's Hospital, Harvard Medical School, Boston, MA, United States

**Keywords:** myeloid neoplasm, childhood, diagnosis, prognosis, treatment

**Editorial on the Research Topic**
Pediatric myeloid neoplasms: new insights into diagnosis, prognosis, and treatment

Unlike adults, childhood myeloid neoplasms are rare and diverse diseases of clonal hematopoietic cell origin. These neoplasms exhibit distinct clinical, morphological, and genetic features that require a specialized pediatric approach (1). These differences also influence diagnostic criteria and disease management, emphasizing the need for tailored recommendations (2).

Among myeloid neoplasms in children, acute myeloid leukemia (AML) is the most common, accounting for approximately 20% of childhood leukemias. However, other myeloid neoplasms, such as myelodysplastic syndrome (MDS), juvenile myelomonocytic leukemia (JMML), chronic myeloid leukemia (CML), and Langerhans cell histiocytosis (LCH), are much rarer, each accounting for less than 5% of pediatric hematologic malignancies. In MDS, the refractory cytopenia of childhood (RCC) subtype is observed in approximately 60% of cases and is a well-recognized form of bone marrow failure, characterized by persistent cytopenia and dysplasia. JMML, on the other hand, is marked by constitutive activation of the RAS signaling transduction pathway. CML is defined by the presence of the *BCR::ABL1* fusion gene, which leads to leukocytosis (3, 4).

This research topic aims to highlight recent advances in the clinical and biological aspects of pediatric patients with myeloid neoplasms. It features four articles: one on LCH and three discussing pediatric AML treatment, novel biomarkers, and recent advances in the literature. Wan et al. reported a case of LCH, a myeloid neoplasm characterized by activating mutations in the mitogen-activated protein kinase (MAPK) pathway. In this study, the patient experienced an unusual accumulation of mononuclear phagocytes infiltrating the stomach, which was initially misdiagnosed as gastric lymphoma.

The diagnosis of pediatric myeloid neoplasms can be challenging due to their heterogeneity and overlapping clinical symptoms with other conditions. Moreover, therapeutic options for most pediatric myeloid neoplasms remain limited. For many of these conditions, allogeneic hematopoietic stem cell transplantation remains the primary, and often the only, potentially curative treatment (4, 5).

Nevertheless, significant progress has been made in the prognosis of AML over the last 25 years, as highlighted by Rao et al. in this research topic. This progress is largely due to the integration of genetic, immunological, transcriptomic, and epigenomic markers. In this context, Bakhtiari et al. utilized a single-cell approach to describe a novel marker, *ARMH1*, for pediatric AML. High *ARMH1* expression was observed in blast cells from patients with disease relapse or a high-risk cytogenetic profile. *ARMH1* expression was associated with poor outcomes and impacted cell proliferation by reducing key cell cycle regulators.

Rao et al. also emphasized, in their bibliometric analysis, that advances in knowledge and treatment are primarily limited to developed countries. The authors noted that global collaboration and the application of advanced tools are essential for personalized medicine and the discovery of new therapeutic targets. Furthermore, Pawiińska-Wąsikowska et al. emphasized the importance of well-assessed clinical features and adequate supportive care for effective clinical management. Their study revealed that treatment-related mortality in AML cases was more strongly associated with hyperleukocytosis, which is particularly valuable in developing countries where new treatments and personalized medicine technologies may be inaccessible due to high costs.

Given the many challenges associated with childhood myeloid neoplasms, continued research is crucial to better understand the unique characteristics of these diseases and ultimately improve patient outcomes. This editorial highlights recent developments in the pathology of these complex neoplasms. By exploring novel discoveries, we hope to foster a deeper understanding of these conditions and drive progress toward more effective diagnostic and therapeutic strategies, specifically for pediatric patients with myeloid neoplasms.
